# KINNTREX: a neural network to unveil protein mechanisms from time-resolved X-ray crystallography

**DOI:** 10.1107/S2052252524002392

**Published:** 2024-04-25

**Authors:** Gabriel Biener, Tek Narsingh Malla, Peter Schwander, Marius Schmidt

**Affiliations:** a Physics Department, University of Wisconsin–Milwaukee, Milwaukee, WI 53211, USA; Harima Institute, Japan

**Keywords:** neural networks, time-resolved X-ray crystallography, machine learning, difference maps, electron density, kinetics, protein mechanisms, loss functions, reaction-rate coefficients, singular value decomposition

## Abstract

Here, a machine-learning method based on a kinetically informed neural network (NN) is introduced. The proposed method is designed to analyze a time series of difference electron-density maps from a time-resolved X-ray crystallographic experiment. The method is named KINNTREX (kinetics-informed NN for time-resolved X-ray crystallography). To validate KINNTREX, multiple realistic scenarios were simulated with increasing levels of complexity. For the simulations, time-resolved X-ray data were generated that mimic data collected from the photocycle of the photoactive yellow protein. KINNTREX only requires the number of intermediates and approximate relaxation times (both obtained from a singular valued decomposition) and does not require an assumption of a candidate mechanism. It successfully predicts a consistent chemical kinetic mechanism, together with difference electron-density maps of the intermediates that appear during the reaction. These features make KINNTREX attractive for tackling a wide range of biomolecular questions. In addition, the versatility of KINNTREX can inspire more NN-based applications to time-resolved data from biological macromolecules obtained by other methods.

## Abstract 2

A kinetics-informed neural-network method (KINNTREX) is designed to analyze a time series of difference maps from a time-resolved X-ray crystallographic experiment.

## 1. Introduction

**Figure 1:**
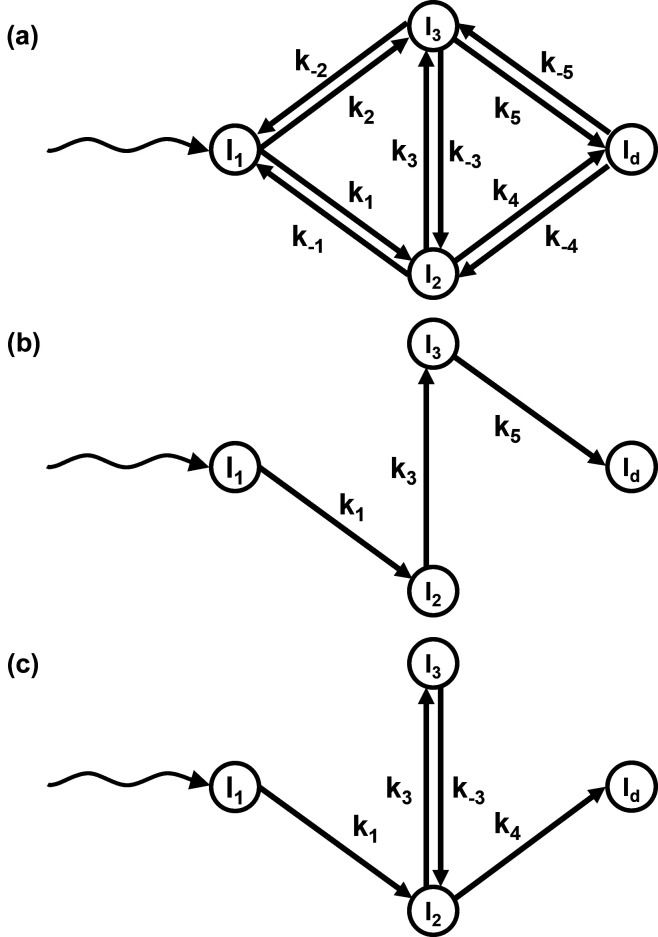
Chemical kinetic mechanisms with three intermediates for (*a*) the general case, (*b*) an irreversible sequential mechanism and (*c*) a dead-end mechanism for a bio-macromolecular reaction initiated by light (wavy arrows). Circles represent intermediate states. The straight arrows labeled with RRCs denote transitions between intermediates.

Biological macromolecules perform essential functions in living organisms. One class of these molecules, proteins, is of particular importance. Proteins are involved in all functions of life, spanning from light perception to the catalysis of essential reactions. To perform their function, proteins must undergo structural (conformational) changes. For example, a catalytically active protein, an enzyme, changes its conformation upon binding of a substrate, during the catalytic conversion of the substrate to product, and when the product leaves (Cornish-Bowden, 2004 ▸). Distinct conformational states along the reaction pathway are called intermediates. The sequence of transitions between intermediates is described by a scheme referred to as a chemical kinetic mechanism (Steinfeld *et al.*, 1999 ▸). Fig. 1 ▸ shows several examples of these mechanisms. Mechanisms may include irreversible processes indicated by single arrows (see Fig. 1 ▸) or reversible processes (arrows pointing in both directions) where an equilibrium between two intermediates is established.

Multiple methods have been developed to determine protein structures with near atomic resolution. X-ray crystallography (Blake *et al.*, 1965 ▸), cryogenic electron microscopy (Yip *et al.*, 2020 ▸) and nuclear magnetic resonance (Wüthrich, 1990 ▸), all offer static snapshots of the protein structure. As a protein changes its structure along the reaction pathway, a single structure is not sufficient to comprehensively describe its function. Time-resolved X-ray crystallography (TRX) aims at determining structure and dynamics at the same time (Moffat, 2001 ▸). TRX captures X-ray diffraction patterns (DPs) during the time the protein performs its function. These DPs are then processed to yield time-dependent electron-density maps (Schmidt, 2019 ▸).

Typically, only a small fraction of molecules in a crystal participates in a reaction because methods to initiate a reaction can be quite ineffective (Srajer & Schmidt, 2017 ▸). As a result, the extent of reaction initiation can be small and of the order of <10%. Conventional electron-density maps are insensitive to the presence of a small admixture of reacting molecules in the presence of a large amount of protein at rest. However, with difference electron density (DED) maps even small amounts of reacting molecules can be detected. A DED map is obtained by subtracting a reference electron density, where all molecules are at rest, from the time-dependent electron density (Henderson & Moffat, 1971 ▸). The DED map has positive and negative features. The negative features are found at locations in the reference structure where an atom has moved away. Positive features are found at positions where the atoms have moved to, or when additional atoms, *e.g.* those of a ligand, bind. The biomolecular reaction is then probed by a time series of difference maps calculated from TRX data, best collected at equidistant time points along logarithmic time (Moffat, 2001 ▸; Schmidt *et al.*, 2003 ▸, 2013 ▸; Rajagopal *et al.*, 2004 ▸; Ihee *et al.*, 2005 ▸; Schotte *et al.*, 2012 ▸).

**Figure 2:**
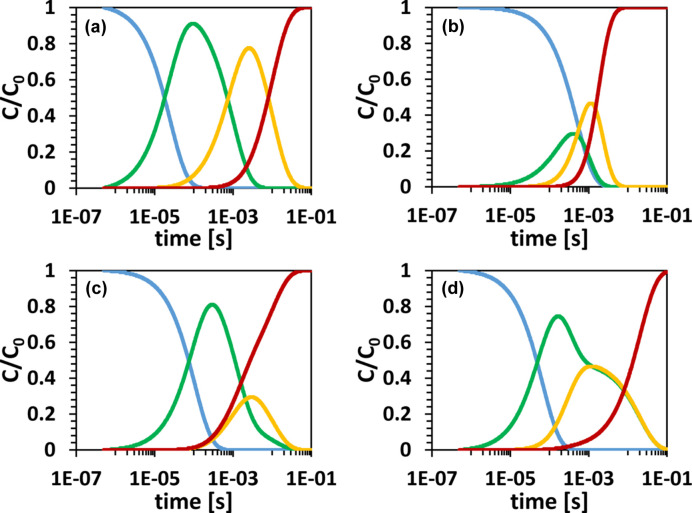
Concentration profiles derived from simulations that mimic the reaction of the blue-light photoreceptor PYP. Fractional concentrations *C*/*C*_0_ are plotted as a function of log *t*. Concentrations of intermediates I_1_ to I_3_ are represented by blue, green and yellow lines, respectively. The concentration of the dark state is represented by the red line. (*a*) The irreversible sequential mechanism with well separated profile peaks. (*b*) The irreversible sequential mechanism with overlapping concentration profiles. (*c*) The dead-end mechanism with separated profile peaks. (*d*) The dead-end mechanism with overlapping concentration profiles. The RRCs for each simulation are summarized in Table 2 ▸. A schematic representation of the irreversible sequential mechanism is shown in Fig. 1 ▸(*b*), and the dead-end mechanism is presented in Fig. 1 ▸(*c*).

The DED map at each measured time point is a sum of all intermediate states, each multiplied by a concentration value at that time point (Moffat, 2001 ▸; Schmidt *et al.*, 2003 ▸; Schmidt, 2023 ▸). The time-dependent concentrations of the intermediates are determined by the chemical kinetic mechanism and the rate coefficients that characterize the transitions between the intermediates (Moffat, 1989 ▸, 2001 ▸). The time evolution of the concentrations of an intermediate during a reaction is called a concentration profile. Fig. 2 ▸ shows such concentration profiles calculated from two mechanisms: the sequential [Fig. 1 ▸(*b*)] and dead-end [Fig. 1 ▸(*c*)] mechanisms. Two sets of reaction-rate coefficients (RRCs) are used for each case. It is evident that at almost all time points the signal is generated by a mixture of species at different concentrations. The concentration profiles are calculated by solving the coupled differential equations of the kinetic mechanism (Steinfeld *et al.*, 1999 ▸).

**Figure 3:**
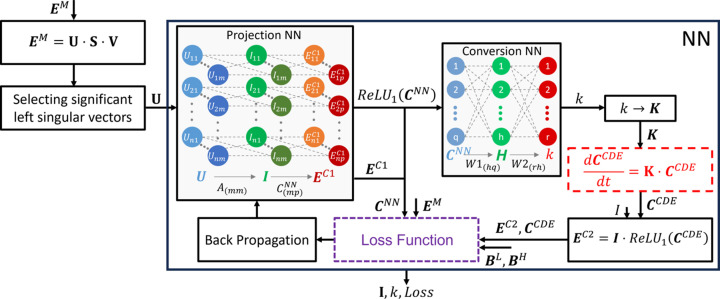
A schematic representation of the analysis method for TRX measurements using KINNTREX. The NN consists of two sub-networks. The first network is called the projection NN. It aims to predict the input, **E** ^M^ (M for measured), as accurately as possible by generating time-dependent DED maps (**E** ^C1^, C for calculated) from significant lSVs (**U**) along with the DED maps of the intermediates (**I**) and the concentrations (**C** ^NN^). The second sub-network called conversion NN takes the **C** ^NN^ as input and predicts RRCs, *k*. **C** ^NN^ is flattened before being applied to the conversion NN. After passing through both sub-networks, KINNTREX solves the differential equation governing the kinetic mechanism of the protein photocycle (red dashed box), resulting in the concentrations **C** ^CDE^. In a subsequent step, prior to the calculation of the loss function, the time-dependent DED maps, **E** ^C2^, are predicted a second time using the DED maps of the intermediates **I** from the projection NN and the **C** ^CDE^ [equation (4)]. The loss function (purple dashed box) evaluates the discrepancies between measured and predicted time-dependent DED maps as well as the differences between the calculated concentrations (**C** ^NN^ and **C** ^CDE^). The user can constrain the adjustable range of the RRCs to further inform the loss function. A backpropagation procedure concludes the NN. The arrows form a loop that iterates multiple times.

To separate the electron density of pure species from a measured time series of electron-density maps, methods from linear algebra are utilized, such as singular value decomposition (SVD) (Schmidt *et al.*, 2003 ▸). SVD has been successfully applied to various time-resolved crystallographic data to (i) determine intermediates structures and (ii) gain information on the chemical kinetic mechanism that involves transformation between intermediates. However, SVD analysis requires expert input. In particular, the chemical kinetic mechanism needs to be estimated. Concentration profiles that are obtained by solving the coupled differential equations of the mechanism are used to obtain the pure intermediate states by using a multi-step procedure (Schmidt *et al.*, 2003 ▸). This procedure (also known as a projection algorithm) is (i) difficult to grasp for the non-expert, (ii) not very user friendly, and (iii) ignores the direct relationship between concentration and (difference) electron density. A neural network (NN) (see Fig. 3 ▸) addresses these challenges since it allows one to restore the relationship between concentration and electron density, and it is user friendly as it does not require the user to understand the mathematics of the projection algorithm.

NNs are artificial-intelligence data-processing algorithms. Inspired by the human brain, NNs are layered structures of artificial ‘neurons’ connected to each other. Each connection is established with a different strength. The variation of the connectivity level across the network provides the ability to learn and establish a reliable output signal. To mimic a neuron in the brain, the artificial neuron is affected by a non-linear activation function. The most commonly used activation function is the so-called rectified linear unit (ReLU) (Zeiler *et al.*, 2013 ▸). NNs are constructed with different architectures: recurrent (recursive) NNs (RNNs) (Medsker & Jain, 2000 ▸), convolutional NNs (CNNs) (Fukushima, 1980 ▸; Gu *et al.*, 2018 ▸), and physically informed NNs (PINNs) and their derivatives (Zaverkin & Kästner, 2020 ▸; Karniadakis *et al.*, 2021 ▸; Ji & Deng, 2021 ▸; Meuwly, 2021 ▸; Westermayr & Marquetand, 2021 ▸; Gusmao *et al.*, 2023 ▸), to name a few. Recently, X-ray crystallography has seen a growth in the use of machine-learning methods and, in particular, NN algorithms for data analysis (Vollmar & Evans, 2021 ▸). Here, an NN as a member of the PINN family of networks is proposed with the goal of extracting DED maps of the intermediates and the corresponding concentration profiles from the time-resolved X-ray data alone. The NN is informed by a system of linear coupled differential equations that describe the kinetic mechanism (Steinfeld *et al.*, 1999 ▸).

## 2. Background and methods

The primary objective is to retrieve structural (conformational) changes in a protein during a reaction. In a time-resolved crystallographic experiment, X-ray DPs are collected at a time Δ*t* after the reaction has been initiated (Moffat, 1989 ▸; Srajer & Schmidt, 2017 ▸). Reflection intensities are extracted from the DPs (Ren *et al.*, 1999 ▸; Schmidt, 2019 ▸), from which structure-factor amplitudes (|*F*_*t*_|) are calculated. Reference structure-factor amplitudes (|*F*_ref_|) are obtained from X-ray data collected on crystals where the molecules are at rest. The structure-factor phases ϕ_ref_ are derived from a well determined reference model. By subtracting the |*F*_ref_| from the |*F*_*t*_|, difference structure-factor amplitudes Δ|*F*|_*t*_ are calculated. With the help of the phases ϕ_ref_, time-dependent DED maps are obtained (Moffat, 1989 ▸; Schmidt, 2023 ▸). The goal is to extract the kinetics from a time series of DED maps. Once this is achieved, time-independent DED maps and ultimately the corresponding structures of the intermediates can be determined. This article introduces a new analysis method to recover the chemical kinetic mechanism and the DED maps of the intermediates. The new analysis utilizes a kinetically informed NN (KINN) specifically designed to work with TRX data. This proposed NN is henceforth named KINNTREX.

### 2.1. Neural-network architecture

The laws of physics are deduced from experimental observations. These laws are described by mathematical formulations that provide predictions for the outcome of experiments not yet conducted. In many cases, the data are so complex that an explicit prediction cannot be obtained. NNs can be employed to resolve such situations. Even though a straightforward theory or mathematical model cannot be constructed, an NN can use existing observations to predict the outcome of experiments yet to be performed.

An elementary unit (a building block) of an NN is a perceptron (also named neuron) (Rosenblatt, 1958 ▸). A perceptron can be expressed mathematically as

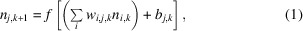

where *n_i, k_* is the value in the *i*th perceptron in layer *k*, *w_i, j, k_* is the weight of the connection between perceptrons *n*_*j*, *k*+1_ and *n*_*i*, *k*_, and *b*_*j*, *k*_ is a bias added before executing the activation function *f*. The activation function is used for changing the perceptron value in a non-linear manner, mimicking a biological neuron (Rosenblatt, 1958 ▸, Block, 1962 ▸). A common NN algorithm starts at the input layer (first layer), propagates through the following layers according to equation (1), and ends at the output layer. This process is called forward propagation. The output of an NN provides the essential missing information, such as the classification of input data (in cases where classification is desired). When an NN is initiated and executed for the first time, the output provides a non-useful relation to the input. This necessitates an iterative training process. Training involves providing inputs along with values that should be predicted by the NN (referred to as ground truths or labels). The output can be compared with the labels using a loss (cost) function, which calculates a loss value. This loss value is utilized to update the weights and biases of the NN, enabling them to be adjusted for improved performance in subsequent iterations. The updating process, known as backpropagation, employs an optimization function such as stochastic gradient descent (Jain *et al.*, 1996 ▸; LeCun *et al.*, 1988 ▸). Such a training procedure is called supervised training. When the training is unsupervised the ground truth is not known. This forces the NN to extract patterns from the input or classify inputs based on differences between them (Hinton *et al.*, 1995 ▸; Chen *et al.*, 2016 ▸). KINNTREX, as introduced here, is an unsupervised NN, which does, however, impose physically meaningful constraints on the data analysis.

#### 2.1.1. Data preparation for KINNTREX

Suppose a time series of DED maps is available from a TRX experiment. Each DED map has been determined at a specific time point Δ*t* after reaction initiation. A DED map consists of a large number of data points (voxels) sampled on a three-dimensional grid, which typically covers one crystallographic unit cell. Each voxel contains a DED value. Structural changes are typically concentrated at a chromophore of a photoactive protein or at the active site of an enzyme where strong DED features are located. Therefore, most parts of the DED maps are free of signal. This allows us to identify a region of interest (ROI) where a strong signal persists. The ROI is carved out from all difference maps in the time series [see Schmidt *et al.* (2003 ▸) for details]. Consequently, the time series contains much less voxels than the original DED maps. The mask used for the selection of the ROI is presented in Fig. S1 of the supporting information, with the atomic structure used for the generation of the mask shown in the inset.

Prior to analysis with the KINNTREX algorithm, the time-dependent ROIs are assembled and organized chronologically based on their acquisition time. Each voxel value of the ROIs is assigned to an element of a column vector. For *N* voxels in the ROI, the column vector is *N* dimensional. This rearrangement is called flattening. The vectors are organized as an *N* × *P* matrix, **E** ^M^ (**E** stands for difference electron density and M for measured), where *P* is the number of time points.

#### 2.1.2. Singular value decomposition of the matrix **E** ^M^

The matrix **E** ^M^ is decomposed into three matrices by SVD:

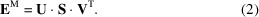

The matrix **U** (*N* × *P*) contains the left singular vectors (lSVs) and the matrix **V** (*P* × *P*) contains the right singular vectors (rSVs). The diagonal matrix **S** (*P* × *P*) includes the singular values along its main diagonal. **V** ^T^ is the transpose of matrix **V**. Matrices **U** and **V** contain significant and insignificant singular vectors, indicated by the diagonal elements of **S**. The insignificant lSVs are discarded and the truncated matrix is used as input to the NN. For time-resolved X-ray data, the selection of significant singular vectors has been discussed in detail in the literature (Schmidt *et al.*, 2003 ▸).

The SVD also allows one to extract relaxation rates. These values are used to constrain the NN, as explained in the following subsections. The relaxation rates are obtained by fitting a sum of exponential functions to the rSVs (Henry & Hofrichter, 1992 ▸; Schmidt *et al.*, 2003 ▸). This can be formulated as follows:

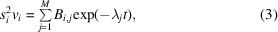

where *s*_*i*_ and *v*_*i*_ are the *i*th singular value and the corresponding rSV, respectively. *B*_*i*, *j*_ is the amplitude of the *j*th process (observed in the *i*th rSV), which must be obtained by a fitting procedure. λ_*j*_ is the *j*th relaxation rate, also calculated in the same fitting procedure, and *t* represents time. Note that λ_*j*_ is globally observed in all significant rSVs. All significant rSVs are fitted simultaneously according to equation (3), using the non-linear least-square fitting Levenberg–Marquardt algorithm (Levenberg, 1944 ▸; Marquardt, 1963 ▸). The minimum number of exponential functions that would be fitted determines the number of distinguishable processes in the reaction, and the minimum number of intermediates, *M* (Rajagopal *et al.*, 2005 ▸; Ihee *et al.*, 2005 ▸).

Once the relaxation rates are obtained, they can be used to define limits for the magnitudes of the RRCs. The RRCs are positive values; therefore, 0 is considered as the lower limit. The upper limit, however, is a multiple of the largest relaxation rate λ_*i*_, throughout this article. An example implementing these constraints is provided in Section 3.4.

#### 2.1.3. Architecture outline

The architecture of KINNTREX is described in Fig. 3 ▸. It consists of two sub-networks, each serving a distinct purpose, as described below. Subsequently, a series of steps are implemented to solve the coupled differential equations that govern the kinetic mechanism of the protein under investigation. These steps inform the NN about the physics of the underlying protein dynamics. The calculation through the NN is repeated for multiple iterations. For each iteration, the loss function is evaluated. The resulting loss value is used to monitor the progress of the calculation. The architecture is outlined by pseudo-code in Appendix *A* [App appa].

#### 2.1.4. First iteration

In the initial iteration, the significant lSVs are loaded into the input layer of the first sub-network, referred to as the projection NN (see Fig. 3 ▸). The projection NN is a so-called partially connected feedforward NN (Kang & Isik, 2005 ▸), as described in Section S2 of the supporting information. In addition to the input layer, the projection NN incorporates a middle layer with perceptrons holding the calculated time-independent DED maps of the intermediates, **I**. The calculation of the intermediates is performed using

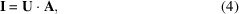

where the matrix **A** contains the weights with which the significant lSVs are multiplied. A third layer within the projection NN is dedicated to the determination of time-dependent DED maps, labeled as **E** ^C1^, as calculated according to

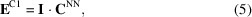

where **C** ^NN^ contains the concentrations of the intermediates. The **C** ^NN^ elements act as weights for the middle-layer perceptrons when calculating the values at the output layer. **I**, **C** ^NN^ and **E** ^C1^ are poorly predicted at this stage as only one iteration has gone through the NN.

Upon passing through the projection NN, the output, **E** ^C1^, is not utilized in the subsequent sub-network. Instead, the **C** ^NN^ modified by the ReLU activation function is loaded into the second sub-network, known as the conversion NN. **E** ^C1^ will be used later to determine a loss value (see below). The conversion NN includes an additional hidden layer, as can be seen in Fig. 3 ▸. This sub-network outputs the RRCs of the chemical kinetic mechanism (see Appendix *D* [App appd]). The number of perceptrons in the hidden layer, *H*, is calculated according to

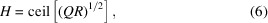

where *Q* = *M* *P*, *M* is the number of intermediates, *P* is the number of time points and *R* is the total number of RRCs within the general mechanism. The term ceil[] rounds the argument to the larger closest integer. The dimensions of required matrices in Fig. 3 ▸ are shown in Table 3 below.

After the two sequential sub-NNs, the coupled differential equations of the chemical kinetic mechanism are solved:

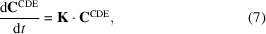

where **C** ^CDE^ represents the time-dependent concentrations (the concentration profiles) of the intermediates and **K** is the coefficient matrix assembled from the RRCs (see Appendix *C* [App appc]). Equation (7) is solved by diagonalizing matrix **K**. The solution of equation (7) is outlined in Appendix *B* [App appb]. Accordingly, the NN is informed by a general chemical kinetic mechanism containing three intermediates plus the reference state. Initial conditions of 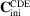 = (1, 0, 0, 0) are applied. Hence, the first intermediate concentration at *t* = 0 was set to 1, while the other two intermediates and the dark-state concentrations were set to 0. The concentrations were normalized to the total number of excited molecules. All these molecules at *t* = 0 can be assumed to be in their first intermediate state. If the non-excited molecules are disregarded, the concentration of the other intermediates as well as the dark state must be 0. The last step that concludes the first iteration through the network recalculates the time-dependent DED maps, **E** ^C2^, using **C** ^CDE^ and **I** calculated in the middle layer of the projection NN, *i.e.*


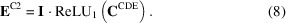

ReLU_1_ is similar to ReLU with the addition of having an upper limit of 1. Hence, values above the limit are set to the limit value. At this point, **E** ^C2^ and **C** ^CDE^ are poorly predicted as only one iteration has been executed.

#### 2.1.5. Iterations through the NN

To monitor the progress of the KINNTREX algorithm, a loss function is used. Only a portion of the resulting loss value is attributed to the comparison between the output of the NN and its input. The loss function is described in detail in Section 2.2. After the loss value is determined, backpropagation is used to minimize the loss by adjusting the weights. Here we choose adaptive moment estimation (AdaM) for optimization of the weights (Kingma & Ba, 2014 ▸). The input to the optimization includes a learning rate that determines how much the weights of the NN are allowed to change. A large learning rate will result in larger change. The computation might converge faster but might converge to a less accurate prediction. For a small learning rate, the opposite is true. Thus, there is an optimal learning rate that can both converge within a reasonable time and provide a correct prediction of the desired information (Bengio, 2012 ▸). Once the loss function has converged to a constant value, the intermediate DED maps, **I**, are obtained along with the RRCs of the chemical kinetic mechanism. This iterative process is both a training procedure and it provides the desired information (concentrations and DED maps of the intermediates).

### 2.2. The loss function

The loss function consists of four parts. These are (1) the time-dependent DED-map loss, *L*_*E*_; (2) the RRC-related loss, *L_K_*; (3) the intermediate-concentration related loss, *L_C_*; and (4) the intermediate DED-maps related loss, *L_I_*. The various parts are described in detail below. The total loss value is calculated as

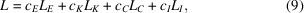

where *c_E_*, *c_K_*, *c_C_* and *c_I_* are the user-specified amplifying factors for the *L_E_*, *L_K_*, *L_C_* and *L_I_* losses, respectively.

#### 2.2.1. Time-dependent DED-map related loss, *L_E_*

The first part, *L*_*E*_, calculates the differences between the input time-dependent DED maps and the two time-dependent DED maps predicted by the NN:

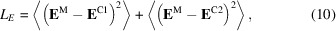

where **E** ^M^ represents the input time-dependent DED maps, and **E** ^C1^ and **E** ^C2^ represent time-dependent DED maps as determined by the NN. The pointed brackets 〈〉 denote averaging over all matrix elements.

#### 2.2.2. Reaction-rate-coefficient related loss, *L_K_*

The second part of the loss function, *L_K_*, constrains the magnitudes of the RRCs (*k_i_*) by allowing them to be within a user-specified range. The loss function penalizes an RRC if it is found outside of the range as follows:

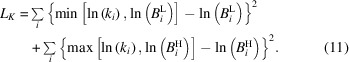

The logarithm has been used because the processes within the chemical kinetic mechanism occur at vastly different rates. This way, fast and slow processes are placed within the same order of magnitude. The min and max functions determine the minimum and maximum values between ln(*k*_*i*_) and the corresponding lower and upper limits, 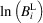 and 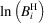 , respectively. An example is shown in Table 1 ▸. When *k*_1_ is between the boundaries, nothing is added to the loss value. When *k*_1_ is out of the range, the loss value will increase. Enforcing the RRCs to stay within the boundaries will decrease *L_k_*. This helps the NN to converge.

#### 2.2.3. Intermediate-concentration related loss, *L_C_*

*L*_*C*_ is determined by the difference between the time-dependent concentration profiles **C** ^NN^ (from the projection NN) and **C** ^CDE^ (from the solution of the coupled differential equations, equation (7)]:

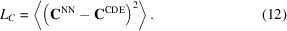

Inclusion of concentration-based loss is imperative for the method to work. This calculation forces the NN towards a self-consistent solution, as will be shown in Section 3.1.

#### 2.2.4. Intermediate DED-maps related loss, *L_I_*

This loss consists of two parts [equation (13)]. The first part (DED_ref_) forces the reference-state DED map to become zero. This part will not be calculated when the DED maps of the intermediates in the projection NN do not include the dark state. If the reference state is included, the target is zero, as the reference DED map (which is featureless) is subtracted by its own prediction. The second (optional) part of this loss value demands that the DED maps of the intermediates be as different as possible from each other. This can be achieved by projecting these DED maps onto each other. The *L_I_* loss value is calculated as

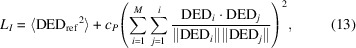

where *M* is the number of intermediates and *c_P_* is a user-selectable amplification factor that weighs the contribution of the projection relative to the rest of the loss value. The similarity (or dissimilarity) is calculated by the dot product between flattened vectors DED_*j*_ and DED_*i*_, where DED_*j*_ and DED_*i*_ represent time-independent DED maps of different intermediates predicted by KINNTREX. If the maps are similar, the dot product is large. This increases the loss value, which the NN intends to minimize. As a result, the NN favors DED maps that are as dissimilar as possible. The double vertical lines (*e.g.* ∥DED_*i*_∥) denote the length of the vector. In this article, *c_P_* has been set to 0, as KINNTREX has converged to a reasonable result. However, in case KINNTREX does not converge, or it converges to a result with undesirable overlap of electron density between intermediates, setting *c_P_* to a value different from 0 might force KINNTREX to output results that are more reliable.

### 2.3. Implementation of KINNTREX

The NN was implemented in Python, with the *PyTorch* package (Paszke *et al.*, 2019 ▸) providing all the necessary tools for the implementation. Unless indicated otherwise, the configuration of the NN was set up as follows: (i) the maximum number of iterations was set to 3 × 10^5^, (ii) the weights and biases were initiated with random numbers (see details in Sections 4.3 and S3), (iii) the learning rate was set to 10^−4^, (iv) the amplifying factors (*c_E_*, *c_K_* and *c_I_*) (Section 2.2) were set to 1 and the factor *c_C_* was set to 0.1, and (v) the (optional) amplifying factor for the projection potion of the intermediate-based loss (*c_P_*) was set to 0. The factor *c_P_* may become useful in future applications of KINNTREX.

KINNTREX reads the ROIs extracted from a time series of crystallographic DED maps. The ROIs are stored in ASCII format in a text file with multiple columns separated by a tab. The first column contains the voxels indexes of the ROI from the crystallographic DED maps. Subsequent columns represent the electron densities of the ROI at subsequent time points.

Apart from the time-dependent DED maps, KINNTREX also requires a model matrix to specify the general kinetic mechanism. An example of this matrix can be found in Appendix *C* [App appc]. Additional inputs include the number of intermediates and a text file with the NN parameters. The critical parameters included in the parameters file are: (i) the standard deviation (STD) of the distribution from which the initial weights are drawn, (ii) the learning rate, (iii) the maximum number of iterations, (iv) the amplifying factors for the different loss-value portions, (v) the loss tolerance and (vi) the boundaries for the RRC ranges. If the loss value is equal to or smaller than the loss tolerance for *n* consecutive iterations, the program finishes. In the case where the loss tolerance is not reached, the algorithm finishes after the maximum number of iterations is reached.

KINNTREX generates the following output files, formatted as text: (i) a list of DED maps of the intermediates after each iteration, (ii) a list of RRCs after each iteration, (iii) the loss value after each iteration and (iv) the predicted concentration profiles of the intermediates after the last iteration. The file containing the DED maps of the intermediates has *M* *N_i_* columns, where *M* is the number of intermediates and *N_i_* is the number of iterations. The file contains *N* lines, where *N* is the number of voxels within the selected ROI. The file containing the RRCs has *R* columns and *N_i_* lines, where *R* is the number of RRCs. The file containing the loss value has a single column with *N_i_* lines. The file containing the concentrations has *M* + 1 columns and *P* lines, where *P* is the number of time points. The concentrations are calculated by solving equation (7) using the RRCs obtained after the last iteration. In this article, the concentration profiles typically contain about 400 time points, to ensure that the plots appear smooth over the entire time range.

In a separate module, crystallographic DED maps of the intermediates are reconstructed by mapping the resulting voxel values back to the ROI of the unit cell. An additional module calculates the residual and weighted residual values for each predicted concentration or time-dependent DED maps and the corresponding ground truth (see Section 2.6). The operations performed by KINNTREX are specified by the pseudo-code listed in Appendices *A* [App appa] [App appb]–*C* [App appc].

#### 2.3.1. Computational performance analysis

The time it takes to analyze DED maps using KINNTREX of course depends on the size of the input data, which can be controlled by the choice of the ROI. However, with resources available nowadays, such as computer clusters, advanced graphical processing units and large amounts of memory, we can mitigate processing-time concerns. For instance, with input data containing 21 time points, about 2500 grid points of DED values (*i.e.* a matrix of 21 × 2500) and 300 000 iterations, the algorithm needed less than an hour to complete, using an Intel Core i7 with 3.4 GHz speed and 7.6 GB RAM. When multiple runs of KINNTREX were required, for example for testing 100 different initial values, a cluster with 100 central processing units was used. Runtime for the complete analysis was again less than an hour.

### 2.4. Ground-truth simulations

The photoactive yellow protein (PYP) has been chosen as a model system in the simulations to test the algorithm. PYP is a photoreceptor found in halophilic bacteria (Meyer *et al.*, 1987 ▸) and it reacts to illumination by blue light (Sprenger *et al.*, 1993 ▸; Hoff *et al.*, 1994 ▸). Once it is excited it undergoes a reversible photocycle. DED maps for 21 different time points were generated by an algorithm published earlier (Schmidt *et al.*, 2003 ▸). Three different intermediates and the dark/reference state were included. The structures of the intermediates were similar but not identical to intermediates I_T_, pR1 and pB determined by TRX (Ihee *et al.*, 2005 ▸; Jung *et al.*, 2013 ▸). Water molecules were removed. Structure factors (SFs) for the dark state, **F**_d_, and the intermediate states, **F**_1_ to **F**_3_, were calculated using the structures of the reference state and the intermediates, respectively.

**Figure 4:**
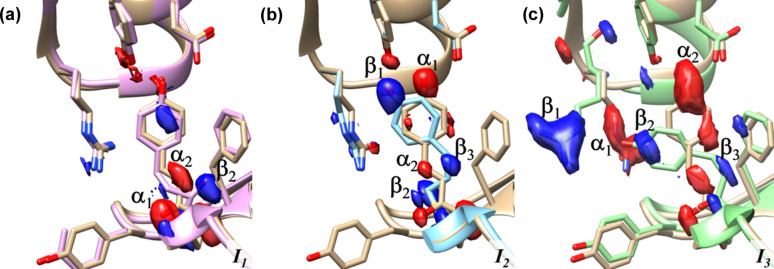
The ground truth. The simulated DED maps are overlaid onto an atomic representation of the (*a*) I_T_, (*b*) pR1 and (*c*) pB intermediate states. Negative electron density is colored red and positive is colored blue. Markings label features in the DED maps. ‘β’ indicates positive DED and ‘α’ indicates negative DED.

The difference SFs (DSFs) of the pure intermediate states have been obtained by subtracting the SFs of the reference state from those of the intermediate states. The three time-independent DED maps shown in Fig. 4 ▸ were calculated from these DSFs. Time-dependent (time resolved) DED maps were calculated from the time-independent maps as shown below. Both simulated time-independent and time-dependent DED maps were taken as the ground truth and could be directly compared to those obtained by KINNTREX.

Two kinetic mechanisms have been tested: the irreversible sequential mechanism [Fig. 1 ▸(*b*)] and the dead-end mechanism [Fig. 1 ▸(*c*)]. Each mechanism was simulated with two different sets of RRCs (see Table 2 ▸). The concentrations of the intermediates at different time points were obtained by solving equation (7) with the selected RRCs as inputs and the initial condition mentioned in Section 2.1.4. The concentration profiles for each simulation are shown in Fig. 2 ▸. The two sequential mechanisms are denoted as S_S_ and S_O_. The concentration profiles for S_S_ (‘S’ for separated) are well separated in time [Fig. 2 ▸(*a*)]. For S_O_ (‘O’ for overlapping), the concentration profile of intermediate I_2_ is buried within that of intermediate I_1_ [Fig. 2 ▸(*b*)]. Two other concentration profiles, DE_S_ and DE_O_, were generated representing the dead-end mechanism. Again, in DE_S_, the intermediate concentrations are separated [Fig. 2 ▸(*c*)]. For DE_O_, the I_3_ concentration profile is fully buried within the I_2_ concentration profile [Fig. 2 ▸(*d*)].

### 2.5. Time-dependent difference maps

The time-dependent SFs were calculated from the SFs of the intermediates **F**_1_, **F**_2_ and **F**_3_ and the dark-state **F**_d_ as follows:

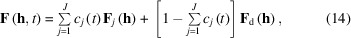

where *c*_*j*_(*t*) is the concentration of the *j* intermediate at time *t*. Noise was added to the amplitudes of the SFs. The noise was estimated from the experimental STDs found in a Laue dataset collected from a PYP dark-state crystal [for details, see Schmidt *et al.* (2003 ▸)]. DSF amplitudes were calculated by subtracting reference state (dark state) SF amplitudes with noise from the noisy time-dependent SF amplitudes. A weight factor calculating the weighted DSF amplitudes has been implemented for the purpose of reducing very large differences resulting from experimental noise (Ren *et al.*, 2001 ▸). With the weighted amplitudes and the phases calculated from the dark-state structural model, time-dependent DED maps were obtained by Fourier transformation using the *CCP4* program ‘*FFT*’ (Winn *et al.*, 2011 ▸). DED features were pronounced near the chromophore region. Accordingly, this region was chosen as the ROI. The ROIs from different time points were assembled into matrix **E** ^M^ and decomposed by SVD. The significant lSVs (columns of matrix **U**) were taken as the input for KINNTREX (Fig. 3 ▸). Table 3 ▸ shows the dimensions of the matrices used by KINNTREX in this study.

### 2.6. Accuracies of KINNTREX

The loss value *L_E_* indicates how close the output time-dependent DED maps are to the input data. The accuracies of the concentrations and those of the time-independent DED maps of the intermediates cannot be estimated, since in an experiment these ground truths are unknown. Optimization of the critical parameters (such as learning rate, maximum number of iterations, boundaries of RRC ranges, *etc*.) can be carried out by using simulated data. In addition, suitable parameters can be found by observing the reproducibility for multiple runs of the NN (see results in Section 4.3). Once the network parameters are found, KINNTREX is primed to work with DED maps of the measured protein of interest.

To compare the concentrations with the ground truth, the weighted residual (*R*_w_) was chosen. *R*_w_ was calculated by using the predicted concentrations and the ground truth (simulated) concentrations as follows:

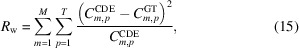

where 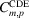 and 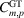 are the predicted and ground-truth concentrations for the intermediate *m* at the time point *p*, respectively. The first summation in equation (15) includes the reference state. Zero values of 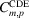 were ignored to avoid making *R*_w_ infinite.

For experimental data, a residual (*R_s_*) can be calculated for the measured and predicted time-dependent DED maps. *R_s_* is similar to the DED-map loss-value portion, *L_E_*. To calculate *R_s_*, we use

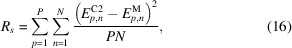

where 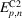 and 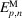 are the predicted and measured time-dependent DED maps, respectively, *N* is the voxel count, and *P* is the number of time points.

To evaluate the reliability of the results, KINNTREX must be executed at least ten times. Each attempt is executed with different random initial values for the weights and biases. The predicted concentrations are extracted from the last iteration at each execution. Once the concentrations are obtained, *R*_w_ and *R_s_* values are calculated using equations (15) and (16), respectively. Following the residual calculations, the values are assembled into histograms. The goodness-of-fit estimation for the data in this article is discussed below (Section 4.3) and elaborated in Section S3.

To assess the accuracy of the DED-map prediction, the Pearson correlation factor (PCF) was determined (Pearson, 1895 ▸). The PCF is calculated as

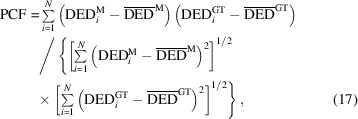

where 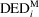 and 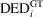 represent the content of the *i*th voxel within the extracted DED map and the ground-truth DED map, respectively, 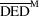 and 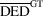 are determined as the average of all voxel values of the corresponding maps, and *N* is the number of voxels common to both maps.

## 3. Results

The performance of KINNTREX was tested with a variety of experimentally realistic scenarios, simulating protein transformation through various chemical kinetic mechanisms. In the first scenario, the NN was tested with data generated by the irreversible sequential mechanism S_S_. In this case, concentration profiles of the intermediates exhibit well separated maximum values [Fig. 2 ▸(*a*)]. For that, KINNTREX was given the following *a priori* information: (i) the number of intermediates obtained after the initial SVD analysis; (ii) a general mechanism containing three intermediates with ten rate coefficients [Fig. 1 ▸(*a*)]; (iii) at *t* = 0, only the first intermediate is populated, all other concentrations are zero; and (iv) all weights and biases of the NN were initialized by drawing random values from a Gaussian distribution with 0 mean and an STD of 0.02. No additional restrictions on rate coefficients were imposed.

In the second scenario, a more challenging irreversible sequential mechanism with overlapping intermediate concentration profiles (S_O_) was tested [see Fig. 2 ▸(*b*)]. To overcome this challenge, the NN was subjected to constraints by limiting values of the RRCs to a certain range [equation (11)]. Relaxation rates obtained from the rSVs were used to determine the limits (see Section 2). The maximum values of all RRCs were set to multiples of the fastest relaxation rate and the minimum values were set to essentially zero. See equation (11) and Table 1 ▸ for examples of their effect on the loss function. This constraint was added to the *a priori* information provided for the former scenario. In fact, this information was provided to KINNTREX when executed for all the scenarios.

In Scenario 3, data simulated from a dead-end mechanism were investigated. This mechanism includes an equilibrium between two intermediates. Such an equilibrium is not present in the irreversible sequential mechanism. The dead-end mechanism is depicted in Fig. 1 ▸(*c*). It introduces another complication as individual concentration profiles can exhibit multiple transient features. Consequently, the multi-featured concentration profile can be misinterpreted as a relaxation of two different intermediates instead of the transition of a single intermediate towards two other intermediates. As an additional challenge to KINNTREX, a severe overlap of two concentration profiles was also introduced in this scenario.

In Scenario 4, a concentration profile with a hardly visible second transient feature was generated [Fig. 2 ▸(*d*)]. This scenario may constitute a significant challenge as the second (weak) transient feature may not be recoverable by KINNTREX.

### 3.1. Scenario 1: the S_S_ mechanism

**Figure 5:**
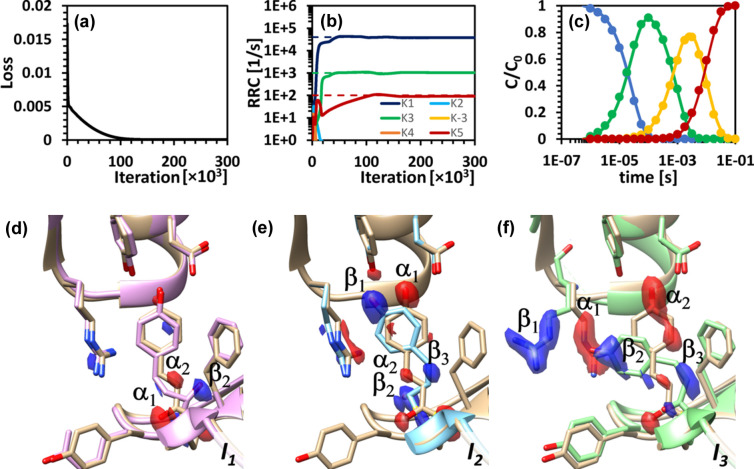
RRCs, concentration profiles and DED maps of the intermediates, as obtained by KINNTREX from time-dependent DED maps generated by the S_S_ mechanism. (*a*) Loss value versus iteration number. (*b*) Predicted RRCs versus iteration number (solid lines), along with the ground-truth values (dashed line). (*c*) Temporal evolution of the relative concentrations of the intermediates at the last iteration (solid lines), along with ground truths (circles). The concentration profiles of intermediates I_1_ to I_3_ are shown by blue, green and yellow lines, respectively, whereas that of the reference-state I_d_ is shown in red. (*d*)–(*f*) DED maps of the intermediates (**I**_1_, **I**_2_ and **I**_3_, as marked in the figure) overlaid on top of their ground-truth atomic structure as well as the reference atomic structure (light brown). Negative electron density is colored red and positive is colored blue.

To test the first scenario (S_S_), KINNTREX was executed with no restrictions imposed upon all ten RRCs of the general mechanism [Fig. 1 ▸(*a*)] (for practical reasons the low and high limits of all RRCs were set to essentially 0 and 10^10^ s^−1^, respectively). Fig. 5 ▸ summarizes the prediction along with the ground truth for the S_S_ simulation. The predicted RRCs [Fig. 5 ▸(*b*) – solid lines] closely align with the ground-truth curves [Fig. 5 ▸(*b*) – dashed lines]. Additionally, the predicted concentrations [Fig. 5 ▸(*c*) – solid lines] exhibit a strong correlation with the ground-truth values [Fig. 5 ▸(*c*) – circles]. The loss value converged close to 7 × 10^−5^ [Fig. 5 ▸(*a*)]

In addition to fully recovering the chemically kinetic mechanism and RRCs, the DED maps of the intermediates [Fig. 5 ▸(*d*)] are very close to the ground-truth DED maps, as shown in Fig. 4 ▸. Both sets of DED maps [from Fig. 4 ▸, and from Figs. 5 ▸(*d*), 5 ▸(*e*) and 5 ▸(*f*)] display negative and positive electron densities at corresponding positions.

This result is truly remarkable as KINNTREX has retrieved the kinetic mechanism along with the intermediate DED maps without any underlying assumptions guiding the analysis. This accomplishment can be attributed to two pivotal factors. First, the concentration values are intricately embedded in the time evolution of the DED maps. The NN capitalizes on its ability to recognize patterns. The pattern recognition is performed by comparing two sets of time-dependent DED maps in the NN: one from the projection NN (**E** ^C1^) and the other calculated by equation (8), following the solution of the coupled differential equations (**E** ^C2^). Both sets of DED maps are compared against the input DED maps (**E** ^M^) and their differences are minimized iteratively. Second, informing the NN by a chemical kinetic mechanism, through coupled differential equations, forces the NN to converge towards the correct answer.

### 3.2. Scenario 2: the S_O_ mechanism

**Figure 6:**
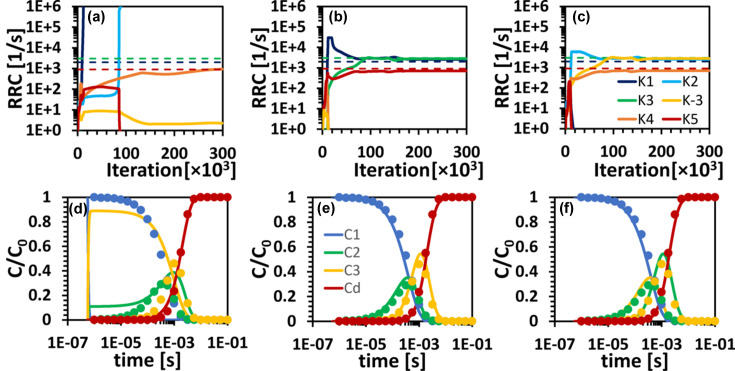
KINNTREX retrieval of the RRCs and the concentration profiles of the intermediates from time-dependent DED maps constrained by three different sets of RRC range limits. (*a*)–(*c*) RRCs versus iteration number for the S_O_ simulation with lower and upper range limits of (*a*) 0 and 10^10^, (*b*) 0 and 3 × 10^4^, and (*c*) 0 and 6000, respectively. The ranges were the same for all ten RRCs. (*d*)–(*f*) Relative concentration profiles of the intermediates for the various simulations as predicted by the NN (solid lines), along with ground truths (circles). Each bottom-panel graph [(*d*), (*e*) and (*f*)] is calculated with the RRCs extracted at the last iteration, as presented in (*a*), (*b*) and (*c*), respectively.

The second scenario involves a more complex irreversible sequential mechanism where two of the concentration profiles overlap [Fig. 2 ▸(*b*)]. The concentration profile of I_2_ is buried in that of I_1_. When the NN is executed with the same constraints as applied in Scenario 1, the prediction is unsatisfactory, as evident in Figs. 6 ▸(*a*) and 6 ▸(*d*). None of the predicted RRCs follow the ground truths at any iteration [Fig. 6 ▸(*a*)]. Apart from the dark-state concentration, none of the intermediate concentrations follow the ground truths. The concentration for the first intermediate is 0 for all time points [Fig. 6 ▸(*d*)]. To improve the prediction, the RRCs were restricted to a range of reasonable values. The upper limit (3 × 10^4^ s^−1^) was set about ten times the highest relaxation-rate value ( 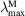 = 2588 s^−1^), the lower limit is essentially zero.

Figs. 6 ▸(*b*) and 6 ▸(*e*) show a significant improvement of the prediction for the RRCs and the concentration profiles, respectively. The RRCs and the concentration profiles (solid lines) coincide well with the ground truths (circles). Further tightening the ranges of the RRCs by a factor of 5 only led to incremental improvement, as illustrated in Figs. 6 ▸(*c*) and 6 ▸(*f*). In Fig. 6 ▸(*f*) the concentration profiles for I_2_ (green) and I_3_ (yellow) are interchanged. Inspection of the mechanism [Fig. 1 ▸(*a*)] reveals that the reaction path from I_1_ to I_3_ and I_2_ is a valid representation of the same sequential mechanism as used for the ground truth.

Using ten adjustable RRCs, the DED maps of the intermediates and the kinetic mechanisms can be predicted correctly without prior knowledge of the exact mechanism. Hence, unlike in the SVD analysis (Schmidt *et al.*, 2003 ▸), where a specific mechanism had to be imposed, KINNTREX eliminates the need for such an assumption.

### 3.3. Scenario 3: the dead-end mechanism

**Figure 7:**
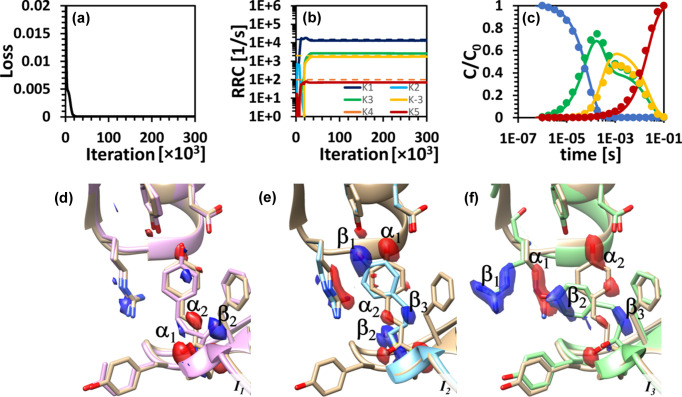
RRCs, concentration profiles and DED maps of the intermediates, as predicted by KINNTREX from time-dependent DED maps for the DE_O_ mechanism. The loss-value calculation includes a comparison between the two calculated concentration profiles, **C** ^NN^ and **C** ^CDE^ (*i.e.* *L_c_*). (*a*) Loss value versus iteration number. (*b*) Predicted RRCs versus iteration number (solid lines), along with the ground-truth values (dashed line). (*c*) Temporal evolution of the relative concentrations of the intermediates as predicted by the NN at the last iteration (solid lines), along with ground truths (circles). The concentration profiles of intermediates I_1_ to I_3_ are shown by blue, green and yellow lines, respectively, whereas that of the reference-state I_d_ is shown in red. (*d*)–(*f*) DED maps of the intermediates (**I**_1_, **I**_2_ and **I**_3_, as marked in the figure) overlaid on top of their atomic structure as well as the reference atomic structure (light brown). Negative electron density is colored red and positive is colored blue. The RRC boundaries were set to essentially 0 and 1.5 × 10^5^ s^−1^ for the lower and upper limits, respectively.

The dead-end mechanism adds another level of complexity due to its reversible process between intermediates I_2_ and I_3_. This mechanism is depicted in Fig. 1 ▸(*c*). DE_O_ exhibits overlapping concentration profiles, as indicated in Fig. 7 ▸(*c*) by the green and yellow points.

The adjustable range limits for all ten RRCs were set to essentially 0 and 1.5 × 10^5^ s^−1^ for the lower and upper limits, respectively. The upper limit is about ten times the value of the largest identifiable relaxation rate ( 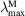 = 14 005 s^−1^).

The lightly restricted NN retrieved both the RRCs [Fig. 7 ▸(*b*)] and the concentrations [Fig. 7 ▸(*c*)], as evident by the overlap of the predicted concentration profiles with those of the ground truths [dashed lines in Fig. 7 ▸(*b*) and circles in Fig. 7 ▸(*c*)]. Again, the loosely restricted NN with no assumptions on the mechanism produced results remarkably close to the ground truth of the mechanism presented in Fig. 9(*b*), despite the complications imposed by the reversible process. The degeneracy between the mechanisms presented in Figs. 9(*b*) and 1 ▸(*c*) (the simulated mechanism) is discussed in Section 4.2.

### 3.4. Scenario 4: the dead-end mechanism – with a small second transient feature along the concentration profile

**Figure 8:**
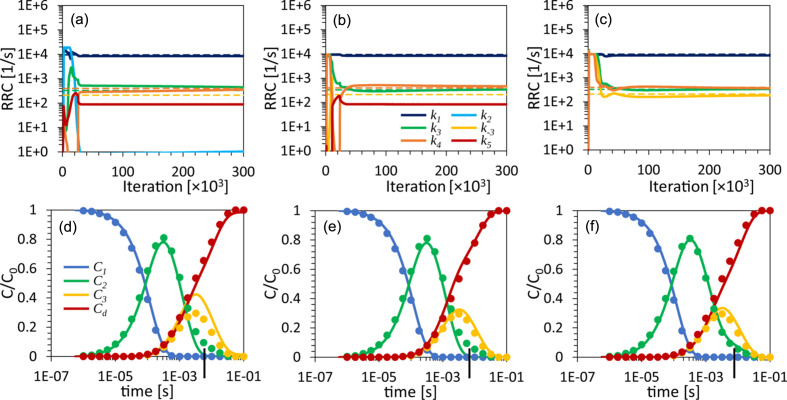
KINNTREX retrieval of the RRCs and the concentration profiles of the intermediates from time-dependent DED maps constrained by three different sets of RRC range limits. (*a*)–(*c*) RRCs versus iteration number for the DE_S_ simulation with lower and upper limits of (*a*) essentially 0 and 9500 for all ten RRCs, (*b*) essentially 0 and 9500 for five RRCs (*k*_1_, *k*_3_, *k*_4_, *k*_5_ and *k*_−3_), and (*c*) 84 and 9500 for four RRCs (*k*_1_, *k*_3_, *k*_4_ and *k*_−3_), respectively. (*d*)–(*f*) Relative concentration profiles of the intermediates for the various simulations as predicted by the NN (solid lines), along with ground truths (circles). Each bottom-panel graph is calculated with the RRCs extracted at the last iteration, as presented in the panels above. The upper limits for the RRCs are in all cases [(*a*)–(*c*)] equal to the sum of the observed relaxation rates, while the lower limit for (*c*) equals the smallest of the relaxation rates. The arrows indicate the weak second transient feature in the concentration profile of I_2_.

Scenario 4 considers data simulated with the DE_S_ mechanism. In this scenario, two complications were introduced: (i) a reversible process between two intermediates, I_2_ and I_3_; and (ii) the second transient feature in the concentration profile of I_2_ buried when substantial noise is added to the simulated data. The results from KINNTREX are summarized in Fig. 8 ▸. The first attempt uses a constrained NN with lower and upper limits set to essentially 0 and 9500 s^−1^ for all ten RRCs [Figs. 8 ▸(*a*) and 8 ▸(*d*)]. The upper limit equals the sum of the relaxation rates obtained from the SVD analysis. This is more restricted than the loose limit of ten times the highest relaxation rate used for Scenarios 2 and 3. As can be seen from Fig. 8 ▸(*a*), a dead-end-like mechanism was not recovered. However, the four RRCs with the most significant magnitudes (*k*_1_, *k*_3_, *k*_4_ and *k*_5_) produced concentration profiles very close to the ground truths. The second transient feature of the I_2_ concentration profile was not recovered [Fig. 8 ▸(*d*), green line and arrow]. In addition, concentrations predicted for intermediate I_3_ are slightly higher than the ground truth [Fig. 8 ▸(*d*), yellow line].

To recover more accurate concentrations, additional constraints were imposed. Five out of the ten RRCs were forced to zero [Figs. 8 ▸(*b*) and 8 ▸(*e*)]. Upper and lower limits of the other five RRCs (*k*_1_, *k*_3_, *k*_4_, *k*_5_ and *k*_−3_) were set close to 0 and 9500 s^−1^, respectively. Still, the obtained concentration profile for intermediate I_2_ did not reveal the second transient feature [see arrow in Fig. 8 ▸(*e*)], but the concentration profile for I_3_ has improved.

In a third attempt, six RRCs were set to zero, with the remaining four RRCs (*k*_1_, *k*_3_, *k*_4_ and *k*_−3_) constrained between 84 and 9500 s^−1^ [Figs. 8 ▸(*c*) and 8 ▸(*f*)], where the lower bound was set to the magnitude of the lowest relaxation rate obtained from the SVD analysis (see Section 2.1.2). The four adjustable RRCs corresponded to those of the dead-end mechanism depicted in Fig. 1 ▸(*c*). This time, the predicted concentration profile for the I_2_ intermediate reveals the second transient feature [arrow in Fig. 8 ▸(*f*)], and all the other predicted concentration profiles agree well with the ground truths. However, in this case, KINNTREX was informed by the underlying mechanism. Unless the mechanism can be identified by other means, small admixtures of I_2_ into I_3_ are unavoidable. In practice, these admixtures may be so small that they remain undetectable in the DED maps and do not affect the recovery of the DED maps of the intermediates in a significant manner.

## 4. Discussion

The RRCs in the KINNTREX algorithm required constraints to provide accurate results. It can be argued that for the simplest scenario involving the irreversible sequential mechanism S_S_, no constraints are needed. For all other scenarios, adding constraints on the *k*s was necessary for accurate results. This is demonstrated below.

### 4.1. KINNTREX overcoming protein kinetics challenges

Several challenges were introduced into the kinetic mechanisms by simulation of different scenarios. These added challenges included: (i) overlapping concentration profiles of the intermediates, (ii) reversible processes and (iii) transient features within a concentration profile that are difficult to detect. While the first two complications were tackled by constraining the RRC ranges relatively relaxed (about ten times the value of the largest relaxation rate), the third complication required a more restrictive approach. Informing KINNTREX with a dead-end mechanism, albeit with unknown reaction rates, was demonstrated in Section 3.4. The dead-end mechanism was informed by forcing six of the ten RRCs to zero. The other four RRCs, which participate in the dead-end mechanism, were constrained to a range. Constraining KINNTREX by informing it with the mechanism is discussed in the following subsection.

#### 4.1.1. Effect of small second transient features on KINNTREX

As shown for Scenario 4 in Section 3, the concentration profile for the I_2_ intermediate contains a second small transient feature (Fig. 8 ▸). Even restricted constraints could not retrieve the concentration profile accurately. Accordingly, several kinetic mechanisms have been selected and tested. A comparison between the attempt to retrieve data by informing KINNTREX with the dead-end and sequential mechanisms is presented in Figs. 8 ▸ and S5, respectively. The sequential mechanism forced seven RRCs to zero, with the remaining three (*k*_1_, *k*_3_ and *k*_5_) constrained between 84 and 9500 s^−1^. Informing KINNTREX with the dead-end mechanism resulted in acceptable predictions. Fig. 8 ▸(*c*) shows overlap between the retrieved RRCs (solid lines) and the ground truths (dashed lines). Fig. 8 ▸(*f*) shows that the predicted concentrations agree closely with the ground truths. However, informing KINNTREX with the irreversible sequential mechanism [Fig. 1 ▸(*b*)] resulted in a failed prediction. As evident in Fig. S5(*a*), only two of the three RRCs in the sequential mechanism were found to be significantly larger than zero. Furthermore, a total mismatch between the retrieved concentration profile (Fig. S5(*b*) solid line) and the ground truth [Fig. S5(*b*) circles] is evident. The loss values calculated for both informed mechanisms can be compared for choosing the appropriate kinetic mechanism. A lower loss value indicates better accuracy. KINNTREX converges to a loss value of 6.4 × 10^−5^ for the dead-end mechanism and 12.9 × 10^−5^ for the sequential mechanism. These values agree with the conclusion from Figs. 8 ▸ and S5 that the dead-end mechanism is the favored one. Furthermore, *R*_w_ for the dead-end mechanism is 0.1 and that for the sequential mechanism is 496 (see Section 2.5 for the estimation of *R*_w_), which clearly favors the dead-end mechanism.

### 4.2. Degenerate chemical kinetic mechanisms

**Figure 9:**
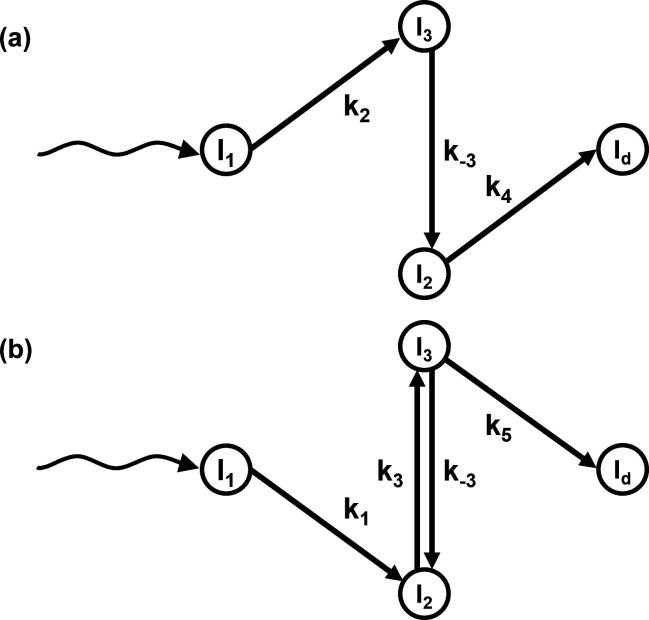
Chemical kinetic mechanisms with three intermediates for (*a*) an irreversible sequential mechanism and (*b*) a sequential mechanism with a reversible process. The wavy arrows represent the light excitation of the protein. Circles represent intermediate states and straight arrows represent transformation between intermediates. The labels on the arrows represent the RRCs.

Typically (except for the S_S_ mechanism), KINNTREX may retrieve a mechanism that differs from the underlying mechanism used in the simulation. One such example is shown in the results presented in Figs. 7 ▸(*b*) and 7 ▸(*c*). In this case, KINNTREX predicts a mechanism that resembles a sequential mechanism with a reversible process between I_2_ and I_3_ as shown in Fig. 9 ▸(*b*), while the data were simulated with the dead-end mechanism depicted in Fig. 1 ▸(*c*). In such a case, the concentration profiles of the intermediates and the corresponding DED maps are essentially identical for both mechanisms. This degeneracy is impossible to break with data collected at a single temperature. Potentially, by varying the temperature as suggested earlier (Schmidt *et al.*, 2010 ▸, 2013 ▸), the degeneracy can be lifted. It is very appealing to implement changes to KINNTREX that can analyze this type of 5D crystallographic data.

### 4.3. Testing the accuracy of the predictions

There is some concern that initiating the NN weights and biases may affect the prediction significantly. To assess this, the NN is randomly initialized with different values for its weights and biases while keeping the rest of the parameters the same. KINNTREX was executed 100 times for each scenario. To speed up the analysis, a computer cluster was utilized with parallel executions. Using the cluster reduced the computational time by two orders of magnitude.

**Figure 10:**
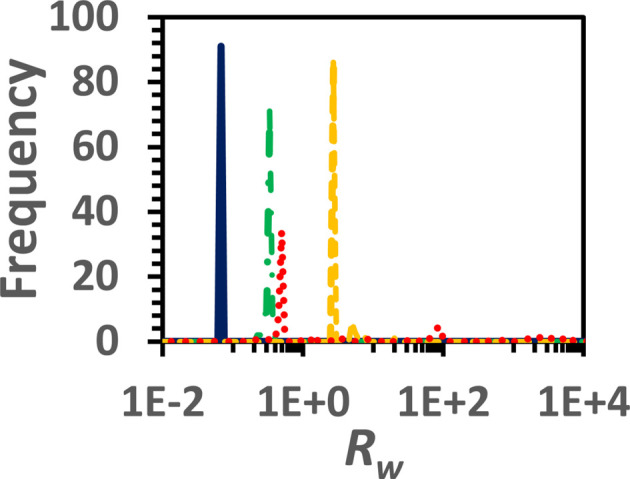
A goodness-of-fit distribution for the S_S_ (blue solid line), S_O_ (green dash-dotted line), DE_S_ (yellow dashed line) and DE_O_ (red dotted line) scenarios described in Section 3. This forms a weighted residual (*R*_w_) histogram calculated between ground-truth and predicted concentrations [equation (15)]. The distributions were assembled from 100 executions of KINNTREX for each scenario, with different initial weights for each execution (see Section 2.5 for the goodness-of-fit description).

The distributions of the weighted residuals [*R*_w_, equation (15)] of the concentrations are narrow (Fig. 10 ▸) no matter how the weights and biases are initiated. The *R*_w_ peak values show that the more complex the data become the higher the peak values (Fig. 10 ▸, blue, green, red and yellow lines for the different scenarios), but the distributions still remain narrow (Fig. 10 ▸). In conclusion, weights and biases do not affect the prediction.

To further assess the accuracy of the prediction, the DED maps extracted by KINNTREX were compared with the ground truth. The PCF was utilized for that comparison (see Section 2.6). The calculated PCF along with the loss values for all the scenarios are summarized in Table S2 of the supporting information. Notably, when the loss value is low (DED shown in Figs. 5 ▸ and 7 ▸) the PCF is higher than 0.97, which indicates high correlation. Conversely, when the loss value is high (DED presented in Fig. S4), the PCF is lower than 0.53 and even becomes negative, indicating low correlation or even anti-correlation with the ground truth.

In a real-world experiment, the ground truths for the concentration profiles are not known. Therefore, the residual, *R*_*s*_, as introduced in Section 2.5, is used. The distributions of the *R*_*s*_ values are, like the *R*_w_ values, also very narrow. Furthermore, the peak values are all ∼3.4 × 10^−5^ (electrons Å^−3^), independent of the complexity of the simulated data. The *R*_*s*_ peak values are comparable to the noise in the input data, which was extracted by reconstructing the data matrix **E** ^M^ without the significant singular vectors and values [equation (2)]. For all scenarios, KINNTREX predicted correct time-dependent DED maps. If the residual is significantly larger than the noise level, KINNTREX’s predictions are most likely inaccurate; see Section S3 for an example.

## 5. Conclusions

Successes of NNs have been demonstrated recently by the popular *AlphaFold2* (Jumper *et al.*, 2021 ▸) and GPT-3 algorithms (Vaswani *et al.*, 2017 ▸). However, GPT-3 and *AlphaFold2* are trained with an enormous amount of data. In contrast, KINNTREX needs only the data from a time-resolved experiment and physically and chemically reasonable constraints. Such physics-informed NNs have been proven to be data efficient, as discussed in the literature (Raissi *et al.*, 2019 ▸).

## 6. Outlook

This article presents the first results employing an NN to analyze time-resolved X-ray data. The NN successfully extracts kinetic mechanisms, time-dependent concentrations and DED maps of intermediate states for several challenging scenarios. More details can be added to improve the performance of KINNTREX, such as forcing the DED maps of the intermediates to be as different from each other as possible (see Section 2.2.4). For example, more hidden layers can be added to the conversion NN shown in Fig. 3 ▸. Furthermore, the linear coupled differential equation solver (shown by the red dashed box in Fig. 3 ▸) can be replaced by a non-linear type. In this way, processes that include higher-order reactions or processes involving diffusion of substrates or ligands (Malla *et al.*, 2023 ▸; Olmos *et al.*, 2018 ▸; Pandey *et al.*, 2021 ▸) could be analyzed as well.

Now, KINNTREX needs to be applied to experimental data. For the benefit of the wider user community, KINNTREX must be equipped with an intuitive graphical user interface. In addition, KINNTREX needs to be extended for reconstruction of the entire unit cell with the predicted DED maps. This requires applying the symmetry operations and periodic boundary conditions, similar to what has been done previously for the application of SVD to TRX data (Schmidt *et al.*, 2003 ▸). The reconstructed content of the unit cell will then be subject to a Fourier transform to obtain DSFs. By extrapolating these DSFs, conventional electron-density maps can be obtained that enables modeling of a structure for each intermediate (Schmidt, 2023 ▸). The implementation will be a formidable challenge for scientists in the years to come.

With some modifications, the proposed method can also be applied to different types of experimental data, such as those obtained by time-resolved small- or wide-angle X-ray scattering (Cammarata *et al.*, 2008 ▸; Cho *et al.*, 2010 ▸; Dmitri & Michel, 2003 ▸; Putnam *et al.*, 2007 ▸), or data obtained by time-resolved absorption spectroscopy. Analyzing time-resolved absorption spectra could be beneficial as measurements are performed on less costly and more accessible instruments. Additionally, such results can be complementary to those obtained from TRX (Zimányi, 2004 ▸; Nagle *et al.*, 1995 ▸).

## 7. Related literature

The following references are only cited in the supporting information for this article: Abraham & Chain (1940 ▸).

## Supplementary Material

Supporting information. DOI: 10.1107/S2052252524002392/it5032sup1.pdf

Supporting code: https://github.com/gabbiener/KINNTREX

## Figures and Tables

**Table 1:** Illustration of the loss value *L*_*K*_ A case where one of the RRCs, *k*_1_, is within the boundaries and another where *k*_1_ is outside the boundary are shown. Sum^L^ and Sum^H^ equal the left and right summation terms in equation (11), respectively. The *L*_*K*_ value for *k*_1_ is calculated in the fourth column. The lower and upper limits, 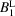 and 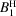 , are set close to 0 [0 is excluded because ln(0) is not defined] and 1000 s^−1^, respectively.

*k*_1_ (s^−1^)	Sum^L^	Sum^H^	Total
900	0	0	0
2000	0	0.48	0.48

**Table 2:** RRCs, with units of per second [1/s], used to generate the concentration profiles in Fig. 2 ▸ The mechanisms and RRCs are also used to simulate ground-truth data for the NN. Based on the mechanism employed and the extent of overlap of the concentrations, the simulations are referred to as S_S_ and S_O_ for sequential separated and sequential overlapped, and DE_S_ and DE_O_ for dead-end separated and dead-end overlapped, respectively.

RRC	S_S_	S_O_	DE_S_	DE_O_
*k*_1_	40000	2000	9500	15000
*k*_3_	1000	3000	330	2000
*k*_4_	–	–	400	100
*k*_5_	100	900	–	–
*k*_−3_	–	–	210	2000

**Table:** Upper part: dimensions of matrices **E**, **U**, **A** and **C** in the projection NN of KINNTREX (see Fig. 3 ▸). In the simulations, *M* = 3 processes are predicted from *P* = 21 time points measured during a reaction, and *N* = 2291 voxels are in the ROI of each DED map. For each matrix, the first subscript denotes the number of rows and the second denotes the number of columns. Lower part: vectors and matrices in the conversion NN. The vector **C** ^NN^ is determined by assembling the rows of matrix **C** ^NN^_(*m*,*p*)_, from the projection NN, into a vector. Matrices **E** ^M^, **E** ^C1^ and **E** ^C2^, as well as **C** ^NN^ and **C** ^CDE^, are comparable and contribute to the loss value.

	Projection NN				
	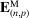	**U**_(*n*,*m*)_	**A**_(*m*,*m*)_	**I**_(*n*,*m*)_	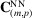
Rows	*N* = 2291	*N* = 2291	*M* = 3	*N* = 2291	*M* = 3
Columns	*P* = 21	*M* = 3	*M* = 3	*M* = 3	*P* = 21

**Table:** 

	Conversion NN				
	**C** ^NN^ †	**W1**_(*h*,*q*)_	**H**	**W2**_(*r*,*h*)_	*k*
Rows	*Q* = *M* *P* = 63	*H* = 25‡	*H* = 25	*R* = 10	*R* = 10
Columns	1	*Q* = 63	1	*H* = 25‡	1

† Converted from **C** ^NN^_(*m*,*p*)_ by subsequently lining up the three rows.

‡ *H* must be greater than *R* [see equation (6)].

## Appendix A Pseudo-code for KINNTREX

This pseudo-code describes the KINNTREX algorithm with the architecture shown in Fig. 3 ▸ (see Section 2.1):

(1) Decompose matrix **E** ^M^ by SVD and estimate the number of significant singular values.

(2) Estimate the relaxation times from the significant rSVs (see Section 2.1.2).

(3) Construct the ‘projection NN’ with an input layer, **U**, a middle layer for intermediate DED maps, **I**, and an output layer for the recalculation of time-dependent DED maps, **E** ^C1^ (see Section S2), with the **C** ^NN^ values as entries of a weight matrix.

(4) Initiate weights matrices between the input and middle layers (**A**), and between the middle and output layers (**C** ^NN^).

(5) Construct the ‘conversion NN’ with a hidden layer, and initiate the weights and biases.

(6) Set constraints for the RRCs using the relaxation times from step (2).

(7) For iteration, *i* ≤ *N*_*i*_.

(8) Calculate **E** ^C1^ along with **I** and **C** ^NN^ using forward propagation of the projection NN.

(9) Calculate the RRC vector, **k**, using forward propagation of the conversion NN with **C** ^NN^ from step (8).

(10) Calculate matrix **K** using the RRC vector according to equations (C3.4) and (C3.5).

(11) Obtain **C** ^CDE^ by solving equation (7) from matrix **K** and the initial condition **C** ^ini^ using the pseudo-code from Appendix *B* [App appb].

(12) Calculate **E** ^C2^ using equation (8), with **I** from step (8) and **C** ^CDE^ from step (11).

(13) Calculate the loss function according to equation (9).

(14) Calculate backpropagation using the optimization method AdaM.

(15) After every *m*th iteration step, save **I**, the RRCs and the loss value. To store the respective values at each iteration, set *m* equal to 1.

(16) End loop.

## Appendix B Pseudo-code for solving differential equations by diagonalization of the **K** matrix

The pseudo-code is as follows:

(1) Diagonalize **K** and find the eigenvalues λ along with corresponding eigenvectors **Λ**.

(2) Calculate  , where λ_*i*_ is the *i*th eigenvalue and *t*_*j*_ is the *j*th time point.

(3) Calculate **Y**(*t* = 0) = **Λ** ^−1^ · **C** ^ini^, where **C** ^ini^ denotes the initial concentrations and **Y**(*t* = 0) is the corresponding value in the diagonalized space.

(4) Calculate  .

(5) Finally, calculate **C** ^CDE^(*t*) = **S** · **Y**(*t*).

## Appendix C Calculating the **K** matrix from RRCs

The dimensions of the matrix **K** are *D* × *D*, with *n*_I_ + 1, where *n*_I_ is the number of intermediates. The addition of 1 accounts for the dark state. To calculate the coefficient matrix **K**, the RRC vector **k** is multiplied by the so-called ‘model matrix’ **M**,

where the vector **a** is later reshaped into the **K** matrix. The vertical dimension,  , of the model matrix is calculated as

The horizontal length,  , equals the dimension of the RRC vector, which is also a function of the number of intermediate states, and is calculated as

In the specific model employed here, it is assumed that intermediate state 1 does not relax directly to the dark state. Vice versa, once the molecules arrive in the dark state, they cannot return to intermediate state 1 without illumination. Consequently, the number *D*_h_ is smaller by two compared with the number calculated from equation (C3.3). If *n*_I_ equals 3, the number of relevant RRCs is equal to 10 [and not 12 as expected from equation (C3.3)]. *D*_h_ = 10 is used here throughout [Fig. 1 ▸(*a*)]. The model matrix **M** used for the simulations on PYP in this article is given by

Finally, the coefficient matrix **K** is obtained by reshaping the vector on the left-hand side of equation (C3.4) [which is vector **a** in equation (C3.1)], into matrix **K** (equation C3.5),

The column sums of matrix **K** are zero. This guarantees that the sum of all concentrations remains constant for all time points. In some cases, certain rate coefficients are constrained to be zero.

## Appendix D Converting from concentrations to RRCs using KINNTREX

The concentration profiles of the intermediates are calculated twice for each iteration. First, they are extracted from the weights in the projection NN. Second, the concentrations are obtained by solving the coupled differential equations of the chemical kinetic mechanism [equation (7)] by diagonalization. The RRCs are contained in the coefficient matrix **K**. To retrieve the RRCs, a simple fully connected NN is used. This network has an input layer that is the flattened concentration vector determined from the projection NN, a hidden layer, and an output layer containing the resulting RRC values. The hidden-layer perceptrons hold the weighted sum of the input values followed by application of an activation function called Leaky-ReLU (Maas *et al.*, 2013 ▸). The output-layer perceptrons hold the weighted sum of the hidden-layer perceptron values, but this time no activation function is applied. This sub-network is part of a larger NN and thus its output is not compared with ground-truth values but instead is used as an input to the calculation of the coupled differential equations. The reason for using Leaky-ReLU is to avoid zero values.
