# Supplementary material for: KINNTREX: a neural network to unveil protein mechanisms from time-resolved X-ray crystallography
Source: IUCrJ. 2024 Apr 25;11(Pt 3):405–22. doi: 10.1107/S2052252524002392 (PMC11067743; doi:10.1107/S2052252524002392)
Supplement: Supplementary file 1 [file m-11-00405-sup1.pdf]

IUCrJ

Volume 11 (2024)

Supporting information for article:

KINNTREX: a neural network to unveil protein mechanisms from time-resolved X-ray crystallography

Gabriel Biener, Tek Narsingh Malla, Peter Schwander and Marius Schmidt

S1. Masking the Region of Interest (ROI).

The region of interest (ROI) was extracted from the difference map using a mask shown in Fig. S1. The mask was generated around a user provided pdb file (Fig. S1, inset). Distances smaller than 3.5 Å from the center of the atoms were considered within the ROI.

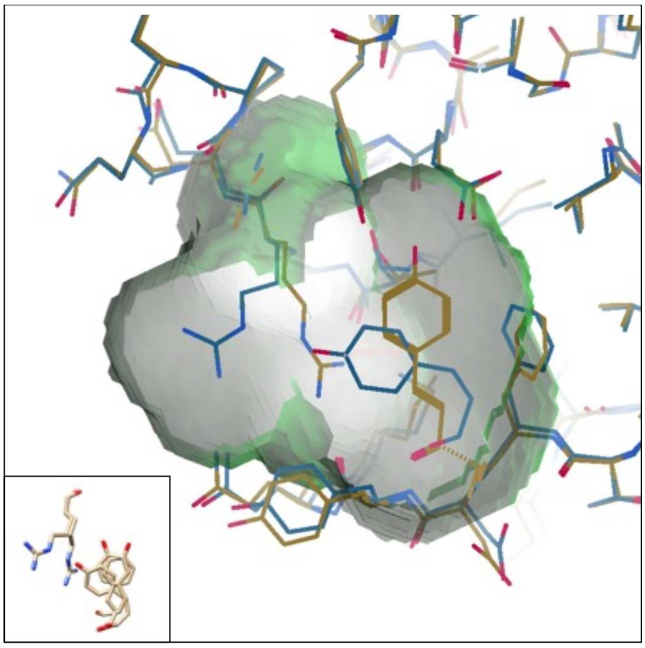

Figure S1 Atomic structure of the PYP in dark state (Orange bond color) along with the atomic structure of the PYP, 2ms after excitation with blue light (Blue bond color). The two atomic structures are overlayed with the mask used throughout the manuscript to calculate the DED maps. **Inset**: atomic structure representation of the residues used for the mask generation.

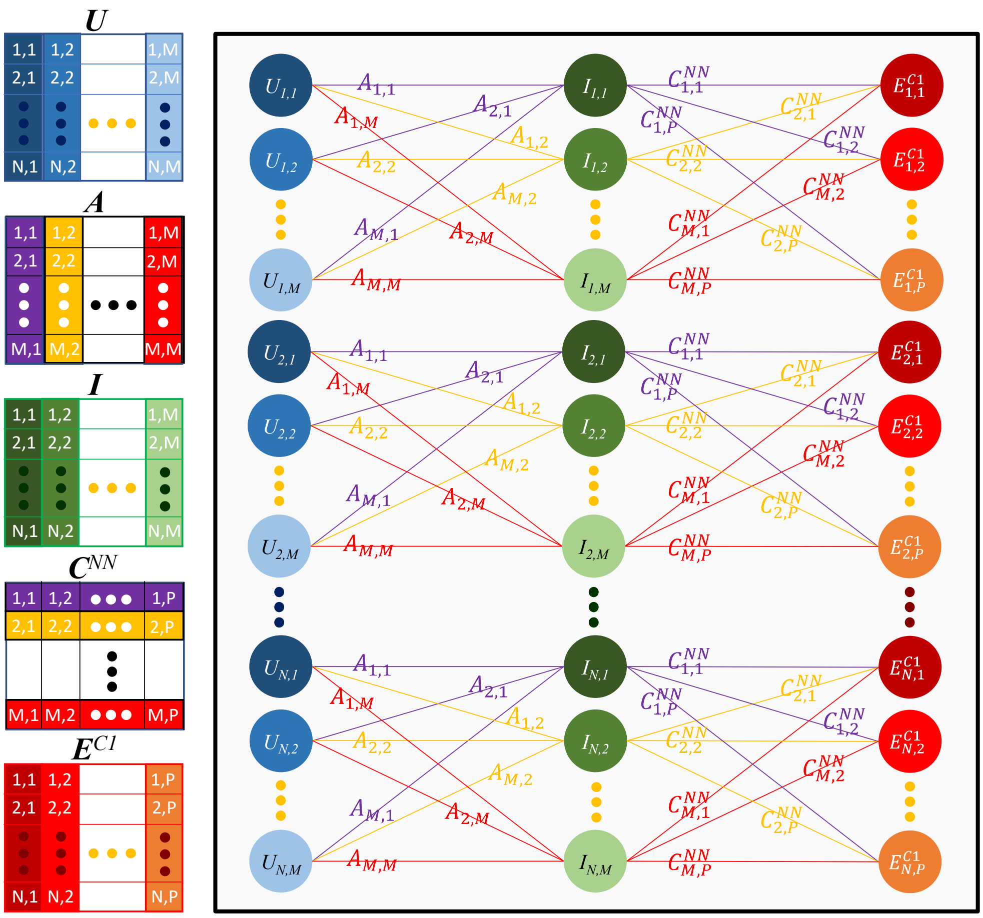

Figure S2 Schematic representation of the Projection NN sub-network. On the right, the network is represented by neurons (circles) and connections (lines) with weights assigned to the connections as indicated by text placed on the connection lines. The first layer **U** is the input layer (blue circle). Time-independent DED maps of the intermediates **I** are obtained in the second layer (green circles), and the red or orange circles represent the time dependent DED maps as an output of the NN. The dimensions of each matrix within the NN is presented in the left side of the figure. The columns of **U** contain the significant left singular vectors. **A** is the projection matrix. **A** is a square matrix containing weights with which the input layer is multiplied. $\mathbf{C}^{\mathrm{NN}}$ is a weights matrix whose rows contain the concentration profiles of the intermediates. Matrix **I** is multiplied with the weights matrix $\mathbf{C}^{\mathrm{NN}}$ to obtain the output matrix (the time-dependent DED maps). Different colors within the $\mathbf{C}^{\mathrm{NN}}$ matrix indicate concentration profiles of different intermediates. Tab, 2 in the main text provides an example for matrix dimensions.

S2. Partially connected neural networks

The input is an $N \times M$ matrix in Fig. S2 labeled **U.** This matrix contains the left singular vectors (lSV). N is the number of grid points in the region of interest (ROI) carved out from the time-dependent DED maps. M is the number of significant lSVs from the SVD analysis, which is also the number of distinguishable intermediates. The matrix **A** can be associated with the projection matrix of the SVD analysis (Abraham & Chain, 1940, Henry & Hofrichter, 1992) or the projection algorithm (Schmidt *et al.*, 2003). Each entry in matrix **A** determines what fraction of an lSV belongs to a particular intermediate. Multiplying **U** by **A** results in an $N \times M$ matrix containing the DED values of the intermediate states. The weights used for the calculation of the output layer are represented by matrix $\mathbf{C}^{NN}$ which is an $M \times P$ array, where P is the number of time points. These weights are equivalent to the time-dependent concentrations of the intermediate (concentration profiles). The resulting time-dependent DED maps are located in the output layer in a $N \times P$ dimensional matrix. The projection NN is partially connected. This scheme can still be considered an NN because of the non-linear activation function (ReLU) applied to $\mathbf{C}^{NN}$. The matrix $\mathbf{C}^{NN}$ is used as the input in the following sub-NN (conversion NN).

S3. Effect of the NN random initiation

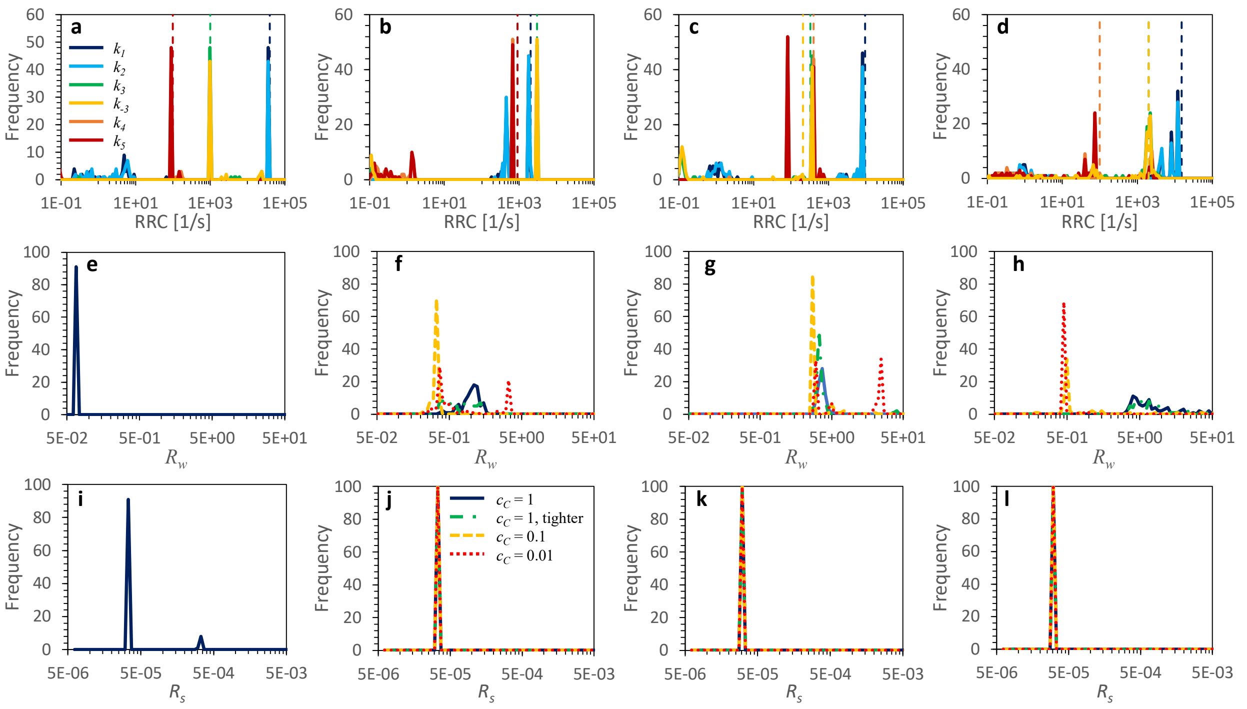

Figure S3 Influence of the amplifying factor c_C for the concentrations in the loss function on the RRCs and the residuals R_w and R_S. Distributions of k_1(dark blue line), k_2 (light blue line), k_3 (green line), k_{-3} (yellow line), k_4 (orange line), and k_5 (red line) for predictions of (**a**) the S_S, (**b**) the S_O, (**c**) the DE_S, and (**d**) the DE_O scenarios. Dashed lines represent the ground truth. The amplifying factor for the concentration (c_C) equals 0.1. (**e-h, middle row**): distributions of R_w for the different scenarios. Each panel corresponds to the panel above. The different conditions include $c_C = 1$ (dark blue line), $c_C = 1$ with tighter constraints on the RRC ranges (green dashed dotted line), $c_C = 0.1$ (yellow dashed line), and $c_C = 0.01$ (red dotted line). (**i-l, bottom row**) Distributions of the residual R_s for the different scenarios. Each panel corresponds to the panels above. The colors of the different c_C in the bottom row of panels are similar to those in the middle row. The distributions were assembled from 100 independent executions of KINNTREX for each scenario with a given c_C.

In KINNTREX the matrices **A**, **W1**, **W2**, $\mathbf{C}^{NN}$, and the biases are initiated with random numbers drawn from a Gaussian distribution with 0 mean and 0.02 standard deviation. The initiation may affect the outcome and thus must be tested. The RRCs were chosen to evaluate the tests along with the figures of merits R_w (Eq. 15) and R_s (Eq. 16). R_w is the weighted residual, calculated between the predicted and ground truth concentrations of the intermediates. R_s is the residual calculated using the input and predicted time-dependent DED maps. In this study, 100 independent executions of the NN were performed for each

scenario S_S, S_O, DE_S and DE_O, respectively. In addition, the amplification factors for the concentrations (c_C, see Eq. 9) were varied from 1.0, 0.1, 0.01 to 0.0. Figure S3 shows the results. The distributions of the RRCs predicted for the S_S scenario (Fig. S3a – solid lines) follow the ground truth (dashed lines) closely. The distributions are narrow indicating that the predictions of the RRCs are reproducible. Hence, the initial values of the weights and biases have little effect on the results. Fig. S3e shows that the peak of the weighted residual R_w is narrowly distributed around 0.07. This value is the lowest among all the scenarios. This result agrees with the precise predictions of the RRCs shown in Fig. S3a. Fig. S3i shows essentially that the same small residual value of $3.48\cdot10^{-5}$ is obtained from all attempts. For the scenarios S_O and DE_O the predictions of the RRCs are presented in Fig. S3b and S3d, respectively. In both cases the predicted RRCs follow the ground truth closely and the distributions are narrow. The distributions of the R_w (Fig. S3f and Fig. S3h) are narrow when the c_C is smaller or equal to 0.1 and the peak positions of the R_w are 0.34 and 0.5,

Table S1 Reaction rate coefficients and total loss values calculated at the last iteration of the NN scheme for the D_O simulation. The table compares between different realizations of the same sample with 8 adjustable reaction rate coefficients and added loss calculation for equating $\mathbf{C}^{NN}$ to $\mathbf{C}^{CDE}$.

	**c_C = 1**	**c_C = 0.1**	**c_C = 0.01**	**c_C = 0**	**c_C = 1, Tight**	**Ground Truth**
k_1	12088	12088	12088	12088	12088	15000
k_2	12088	12088	12088	12088	12088	0
k_3	3641	2208	1998	4024	3641	2000
k_4	49	74	81	49	49	100
k_5	45	74	81	49	45	0
k_{-1}	0	0	0	0	0	0
k_{-2}	0	0	0	0	0	0
k_{-3}	3641	2208	2208	4024	3641	2000
k_4	1	0	0	2	1	0
k_5	1	0	0	2	1	0
R_w	4	0.5	0.45	735	5	
R_s	$3.3\cdot10^{-5}$	$3.3\cdot10^{-5}$	$3.3\cdot10^{-5}$	$9\cdot10^{-5}$	$3.3\cdot10^{-5}$	Noise=$3.2\cdot10^{-5}$

respectively for the two scenarios. For the $c_C > 0.1$ the R_w peaks are above 0.45 and 4, respectively for the different scenarios and are more spread. Wider distribution indicates a lack of reproducibility. For $c_c = 0.1$ we get the best results with our type of data for all scenarios. For KINNTREX the initiation of other weights and biases does not play an important role since the distributions are small. Since the R_w is not accessible to real data, an optimum c_c may be determined with simulated data using the protein under investigation and a suitable ROI as input to KINNTREX.

As indicated by Tab. S1 for the DE_O scenario the only RRCs that should have values different from zero are k_1, k_3, k_{-3}, and k_4. However, predictions have resulted in other RRCs with much larger values such as k_2 equals 12088 and k_5 has values equal and higher than 45. Tab. S1 shows results from a statistical analysis of 100 executions of KINNTREX for each case. Hence, in some instances the kinetic mechanism follows a sequence shown in scheme S1a while others follow a sequence presented in scheme S1b. Obviously, both schemes represent the same mechanism (Fig 1a). KINNTREX cannot distinguish between these two schemes and randomly chooses either the one or the other. In both cases the concentration profiles as well as the DED maps of the intermediates are predicted correctly.

$$I_1 \overset{k_1}{\rightarrow} I_2 \underset{k_{-3}}{\overset{k_3}{\leftrightarrow}} I_3 \overset{k_4}{\rightarrow} I_d \quad \text{(S1a)}$$

$$I_1 \overset{k_2}{\rightarrow} I_3 \underset{k_3}{\overset{k_{-3}}{\longleftrightarrow}} I_2 \overset{k_5}{\rightarrow} I_d \quad \text{(S1b)}$$

KINNTREX was initiated with weights and biases values drawn from a uniform distribution set between -1 and 1. This attempt was tested using scenario S_O. The results were not different from the ones using Gaussian distribution with a standard deviation of 0.02 centered around 0.

S4. Ignoring the comparison between C^{NN} and C^{CDE} in the loss function by setting c_c = 0

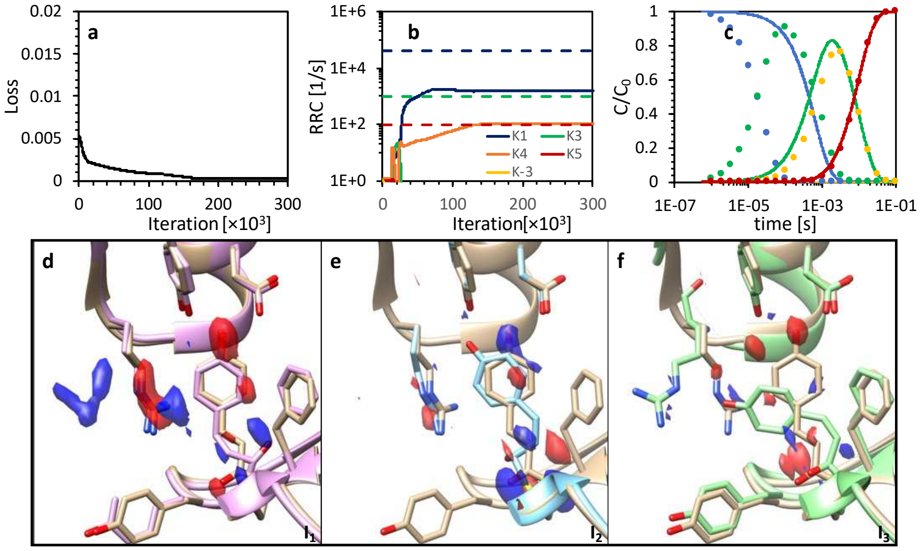

Figure S4 RRCs, concentration profiles and DED maps of the intermediates as predicted by the NN from time dependent DED maps for the S_S mechanism. The loss value calculation exclude comparison between two calculated concentration profiles, $\boldsymbol{C}^{NN}$ and $\boldsymbol{C}^{CDE}$. (**a**) Loss value vs iteration number. (**b**) predicted RRCs vs iteration number along with the ground truth value (dashed line). (**c**) Temporal evolution of the relative concentrations of the intermediates as predicted by the NN at the last iteration (solid lines) along with ground truth (circles). C1 to C3, Cd in the figure legend represent the concentrations at intermediates I_1 to I_3 and I_d, respectively, where I_d is the reference (dark) state. (**d**) DED maps of the intermediates (**I_1**, **I_2**, and **I_3** as marked in the figure) overlaid on top of their atomic structure as well as the reference atomic structure (light brown). Negative electron density is colored red and positive colored green. The RRC boundaries were set to 0 and 10^{10} for the lower and upper limits, respectively.

When the c_C equals zero, KINNTREX is executed without the information on the kinetic mechanism. KINNTREX is unable to predict sensible concentration profiles and DED maps (Tab. S1 and Fig. S4). R_w is 4 orders of magnitude larger than for the other cases, and R_s is about 3 times higher than the noise level while the other cases have values very close to the noise level. KINNTREX predicts 2 RRC when 3 are required (Fig. S4b). Fig. S4c shows a total mismatch between the predicted and the ground truth concentration profiles. The comparison between $\boldsymbol{C}^{NN}$ and $\boldsymbol{C}^{CDE}$ is imperative. Already the smallest weight c_C = 0.01 has been sufficient for a meaningful prediction.

S5. Effect of setting individual RRCs to zero

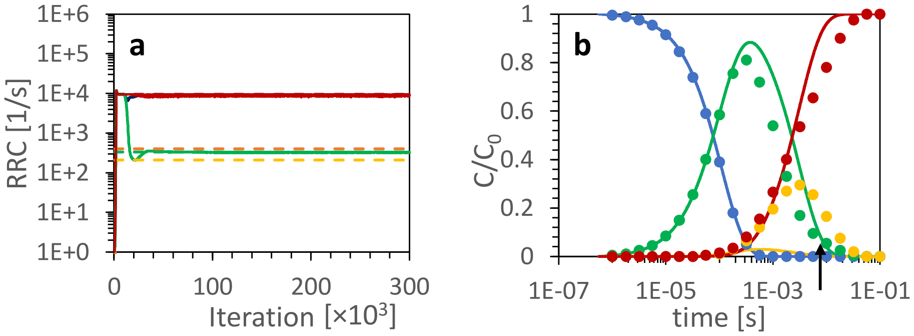

Figure S5 KINNTREX predictions of the RRCs and the concentration profiles of the intermediates retrieved from time- dependent DED maps. (**a**) RRCs vs iteration number for the DE$_S$ simulation with lower and upper range limits of 84 and 9500 for 3 RRCs (k_1, k_3, and k_5), respectively. predicted RRCs include k_1 (dark blue solid line), k_3 (green solid line) and k_5 (red solid line). Ground truth RRCs include k_1 (dark blue dashed line), k_3 (green dashed line), k_{-3} (yellow dashed line), and k_4 (orange dashed line). (**b**) Relative predicted concentration profiles of the intermediates (C_1 – blue solid lines, C_2 – green solid line, C_3- yellow solid line, and C_d – red solid line) for the candidate presented in (**a**) along with ground truth (circles). The colors of the ground truth concentration profiles match the corresponding colors of the predicted concentration profiles. The concentrations are calculated with the RRCs extracted from the last iteration. The upper limits for the RRCs were set to the sum of the relaxation rates, while the lower limit was set to the minimum of the relaxation rates. The arrow in (**b**) indicates the second transient along the concentration profile of I_2 for the simulated dead-end mechanism.

Setting individual RRCs to zero during the execution of KINNTREX is equivalent to enforcing a specific candidate chemical, kinetic mechanism from the general mechanism. KINNTREX is then informed with the candidate mechanism with relevant RRCs and unknown magnitudes. Fig 8 shows the result when NN is informed with a correct candidate, in this case the dead-end mechanism. Fig. S5 presents the prediction of KINNTREX when it was informed with the sequential mechanism, but the simulated input data correspond to the dead-end mechanism. The prediction is very poor. Fig. S5a shows that only two of the three RRC in the sequential mechanism were predicted to be different from zero. Fig. S5b shows the mismatch between the predicted and ground truth concentration profiles (R_w = 496). Since with experimental data R_w cannot be evaluated, the R_s needs to be calculated and compared when various candidate mechanisms are employed. The candidate with the lowest R_s needs to be selected. In general, candidates can be selected to explicitly test and refine several scenarios, but in the general case, this is not necessary.

S6. Comparing DED maps extracted by KINNTREX to the ground truth with PCF.

Table S2 Loss value and Pearson correlation factor (PCF) calculated for predicted DED maps extracted using KINNTREX for different simulated mechanisms. Predicted maps were compared to the ground truth shown in Fig. 4 of the main text. SEQ 1 is the mechanism simulated in scenario 1 in the main text, SEQ 2 is mechanism simulated in scenario 2 DE 1 is the mechanism simulated in scenario 4 and DE 2 is the mechanism simulated in scenario 3.

Mechanism	Loss Value	Prediction Quality	Interm.	PCF	DED Map
SEQ 1	6.88×10^{-5}	High	1	0.996	Fig. 5a
			2	0.9965	Fig. 5b
			3	0.9816	Fig. 5c
SEQ 2	7.17×10^{-5}	High	1	0.996	Fig. 6b
			2	0.969	DED map not included
			3	0.976	
DE 1	6.53×10^{-5}	High	1	0.996	Fig. 8a
			2	0.996	DED map not included
			3	0.977	
DE 2	6.53×10^{-5}	High	1	0.996	Fig. 7a
			2	0.993	Fig. 7b
			3	0.977	Fig. 7c
SEQ 1*	3.75×10^{-4}	Poor	1	0.418	Fig. S4a
			2	-0.664	Fig. S4b
			3	0.531	Fig. S4c

* The loss value calculation exclude comparison between two calculated concentration profiles, $\boldsymbol{C}^{NN}$ and $\boldsymbol{C}^{CDE}$.

S7. Supplementary References

Abraham, E. P. & Chain, E. (1940). *Nature* **146**, 837.

Henry, E. R. & Hofrichter, J. (1992). *Method Enzymol* **210**, 129-192.

Schmidt, M., Rajagopal, S., Ren, Z. & Moffat, K. (2003). *Biophysical journal* **84**, 2112-2129.
